# Early Pain Exposure Influences Functional Brain Connectivity in Very Preterm Neonates

**DOI:** 10.3389/fnins.2019.00899

**Published:** 2019-08-23

**Authors:** Domenico Tortora, Mariasavina Severino, Carlo Di Biase, Maryia Malova, Alessandro Parodi, Diego Minghetti, Cristina Traggiai, Sara Uccella, Luca Boeri, Giovanni Morana, Andrea Rossi, Luca Antonio Ramenghi

**Affiliations:** ^1^Neuroradiology Unit, IRCCS Istituto Giannina Gaslini, Genoa, Italy; ^2^Neonatal Intensive Care Unit, IRCCS Istituto Giannina Gaslini, Genoa, Italy; ^3^Child Neuropsychiatry Unit, IRCCS Istituto Giannina Gaslini, Genoa, Italy

**Keywords:** preterm neonates, fMRI, pain, functional connectivity, neonatal neuroimaging, nociceptive modulations, brain connectivity, resting state

## Abstract

**Background:**

Early exposure to nociceptive events may cause brain structural alterations in preterm neonates, with long-lasting consequences on neurodevelopmental outcome. Little is known on the extent to which early pain may affect brain connectivity. We aim to evaluate brain functional connectivity changes in preterm neonate that underwent multiple invasive procedures during the postnatal period, and to correlate them with the neurodevelopmental outcome at 24 months.

**Methods:**

In this prospective case-control study, we collected information about exposure to painful events during the early postnatal period and resting-state BOLD-fMRI data at term equivalent age from two groups of preterm neonate: 33 subjected to painful procedures during the neonatal intensive care (mean gestational age 27.9 ± 1.8 weeks) and 13 who did not require invasive procedures (average gestational age 31.2 ± 2.1 weeks). A data-driven principal-component-based multivariate pattern analysis (MVPA) was used to investigate the effect of early pain exposure on brain functional connectivity, and the relationship between connectivity changes and neurodevelopmental outcome at 24 months, assessed with Griffiths, Developmental Scale-Revised: 0–2.

**Results:**

Early pain was associated with decreased functional connectivity between thalami and bilateral somatosensory cortex, and between the right insular cortex and ipsilateral amygdala and hippocampal regions, with a more evident effect in preterm neonate undergoing more invasive procedures. Functional connectivity of the right thalamocortical pathway was related to neuromotor outcome at 24 months (*P* = 0.003).

**Conclusion:**

Early exposure to pain is associated with abnormal functional connectivity of developing networks involved in the modulation of noxious stimuli in preterm neonate, contributing to the neurodevelopmental consequence of preterm birth.

## Introduction

The survival rates for preterm infants have improved considerably in recent decades due to the advances in perinatal and neonatal care (Lea et al., 2017). On the other hand, preterm neonates necessitate invasive treatments as part of their essential intensive care and can be subjected to multiple painful procedures requiring skin breaks (i.e., heel lances, intravenous or central line insertion, intramuscular injection, and surgical interventions) or intubations that may have a relevant influence on normal brain maturation and developmental neuroplasticity especially at low gestational ages (Carbajal et al., 2008). Indeed, in the early phases of development, the brain is extremely adaptable to novel sensory information inducing experience-dependent plasticity mechanisms (i.e., adaptive neuroplasticity) and vulnerable to several different insults leading to reactive post-injury neuroplasticity (Ismail et al., 2017).

In this regards, it has been recently demonstrated that exposure to nociceptive stimuli at earlier gestational ages may have several effects on the developing brain, with more severe alterations of multiple cerebral structures in extremely preterm neonates exposed to multiple painful procedures (≤28 weeks of gestational age) (Brummelte et al., 2015; Duerden et al., 2018). In particular, pain in preterm neonates may induce somatosensory changes (Grunau et al., 1994; Goffaux et al., 2008; Walker et al., 2009a), with long-lasting consequences upon subcortical gray and white-matter development subserving different somatosensory, cognitive, and motor processes (Brummelte et al., 2012; Ranger et al., 2013).

In preterm neonates, altered brain structure contributes to poorer cognitive, and motor outcomes during the first years of life (Grunau et al., 2009; Brummelte et al., 2012; Vinall et al., 2014; Duerden et al., 2018).

Despite the increasing evidence that repeated painful experiences during the first days of life in the neonatal intensive care environment may contribute to structural alterations of the preterm neonatal brain, little research has focused on the relationship between painful stressors and functional activity of the preterm brain at term corrected age (Smith et al., 2011). Recent studies demonstrated weaker functional connectivity in preterm neonates evaluated at term corrected age, with more severe alterations of network complexity in neonates with white matter injuries (Ball et al., 2016; Scheinost et al., 2016; Duerden et al., 2019). Nevertheless, little is known on the extent to which painful stressors may affect brain connectivity in these early phases, with potential consequences on future brain development, and plasticity.

In this study, we hypothesized that early exposure of preterm infants to painful stimuli in the neonatal intensive care setting could influence the functional connectivity of brain regions involved into pain processing. Accordingly, we aimed to explore differences in functional brain connectivity among preterm neonates that underwent multiple invasive procedures during the perinatal period and a control group of gestational-age and sex-matched neonates who experienced less than 5 procedures and no intubation nor surgical interventions. Additionally, we aimed to assess if abnormalities of brain connectivity are related to the invasiveness of treatments, correlating brain connectivity features with the number, and type of painful events. Finally, we aimed to explore the association between functional connectivity alterations at term-corrected age in preterm neonates both exposed and not exposed to painful stimuli and their neurodevelopmental outcome at 24 months.

## Materials and Methods

The local ethics committee (Comitato Etico Regione Liguria, Genoa, Italy) approved this study and parents provided written informed consent in accordance with the Declaration of Helsinki.

### Participants

We retrospectively identified all 112 preterm neonates with birth weight <1500 g who underwent brain MRI at term equivalent age within our institutional screening program for identification of prematurity-related lesions between July 2016 and January 2017. Clinical data were systematically collected from the neonates’ charts by a neonatologist. As a measure of pain, each individual invasive procedure involving skin breaks was considered, including heel lances, intubations, intravenous or central line insertion, intramuscular injection, and surgical interventions. Other clinical variables included gestational age at birth, postmenstrual age at MRI scan, gender, APGAR scores, morphine dose, midazolam dose, dexamethasone dose, and days of mechanical ventilation.

Neonates were first divided into two groups: (i) those who were subjected to invasive procedures during the neonatal period (IP-group) and (ii) those who were not subjected to invasive procedures (control group). Subsequently, we elaborated a neonatal intensive care invasiveness (NICI) score based on the type and number of invasive procedures: 0, no painful events during the early post-natal period or <5 skin breaks (i.e., heel lances, central line insertion, and intramuscular injection); I, >5 skin breaks or neonatal endotracheal intubation; II, >5 skin breaks and neonatal endotracheal intubation; III, surgical interventions (i.e., patent ductus arteriosus ligation and surgical necrotizing enterocolitis). Each neonate was assigned to one of these categories by the neonatologist (AP) who assisted all the infants during the postnatal period.

Exclusion criteria were: (i) presence of significant cerebral injuries (congenital malformations/syndromes, antenatal infections, ischemic lesions, hemorrhagic infarctions, or intraventricular-germinal matrix hemorrhage), (ii) absence of resting state fMRI in the study protocol, and (iii) poor quality of MR images due to motion artifacts.

### Neurodevelopmental Outcome

The neurodevelopmental assessment was performed at 24 months of age between July 2018 and January 2019, using the Griffiths Mental Development Scales-Revised: Birth to 2 years (Griffiths et al., 2007). Developmental quotient, locomotor, personal-social, hearing-speech, eye-hand coordination, and performance outcomes subscales were assessed by 2 testers (LB and SU), unaware of brain imaging findings (Griffiths et al., 2007).

### Brain MRI

MR imaging was performed on a 3T Philips MR scanner (Ingenia 3T; Philips Healthcare, Best, Netherlands) using a 32-channel parallel imaging head coil. Anatomical MR imaging was acquired with a high-resolution 3D T1-weighted image sequence (1 mm^3^ × 1 mm^3^ × 1 mm^3^ voxel size). For each subject we performed a resting state scan consisting of 250 volumes (5 min and 45 s), using an echo-planar imaging with TR/TE = 2500/30 ms, flip angle = 90°, 53 slices, 96 × 96 matrix size, FOV = 288 mm × 288 mm, voxel size = 3 mm isotropic, and an interleaved mode of slice acquisition. Volumetric T1-weighted sequence, axial and coronal T2-weighted sequences and susceptibility-weighted imaging (SWI) scans were also evaluated for brain lesions.

All patients were fed before MRI examination in order to achieve spontaneous sleep and were spontaneously breathing during examination. Hearing protection was used in all patients. Heart rate and oxygen saturation were non-invasively monitored by pulse-oximetry during examination.

### Structural Analysis

Brain segmentation was performed on 3D T1-weighted sequences calculating gray matter, white matter, and cerebrospinal fluid maps. Non-brain tissue components were removed by using Skull stripping toolbox of BrainSuite version 15c^1^. Extracted images were then segmented into tissue classes using unified segmentation as implemented in the “Segment” option of SPM12 (Wellcome Department, University College London, United Kingdom). For guiding segmentation, we used tissue probability maps from preterm neonates scanned at term age (Kuklisova-Murgasova et al., 2011). A neuroradiologist with 16 years of experience (GM) validated brain segmentation results by qualitative assessment.

### Resting State Analysis

Prior to functional data analysis, every BOLD sequence was preprocessed using SPM12 software (Welcome Trust Center of Neuroimaging, University College London, United Kingdom). The first four scans were not considered for the analysis. Image preprocessing included slice-time corrections, image realignment to median image of each sequence, co-registration of functional images and structural 3D T1-weighted images, and normalization of structural and functional scans to a neonatal template (Shi et al., 2011). Functional volumes were spatially smoothed using an 8 mm FWHM Gaussian kernel to address the anatomical variability that characterizes the neonatal brain. Subject level connectivity analysis was performed using the Conn toolbox v18b (Whitfield-Gabrieli and Nieto-Castanon, 2012) on Matlab. Functional volumes were band pass filtered at default values of 0.008–0.09 Hz simultaneously with nuisance regression. Subject specific nuisance regressors included 6 movement regressors and their time derivatives, and 5 regressors pertaining to white matter and CSF signals sources, respectively, using a principal component (PCA) based noise correction approach (Behzadi et al., 2007).

Differences in functional connectivity among the two groups of preterm neonates were investigated using a data-driven principal component based multivariate pattern analysis (MVPA)^2^. The MVPA provides a regionally unbiased mapping of brain areas that show abnormal whole brain connectivity patterns according to the exposure to early procedural pain events. In particular, we tested for clusters of different brain connectivity that differed between neonates with exposure to early procedural pain and controls, as represented by PCA component volumes. For the analysis we considered 8 PCA components (*C* = 8) and we maintained the four principal components that explained most of the variance of the connectivity matrix (Thompson et al., 2016). Subsequently, we performed a whole-brain *post hoc* seed correlation analysis using spherical seeds placed at the peak voxels of the major clusters obtained in the MVPA, in order to investigate differences in connectivity patterns of these regions against all other voxels in the brain. This latter *post hoc* analysis aims to characterize the observed patterns of connectivity between the found MVPA seeds and the rest of the brain, testing which areas of the brain changes its connectivity in relation to exposure to procedural pain. Additionally, a seed-to-voxel analysis, considering the same seeds placed at the peak voxels of the major clusters obtained in the MVPA, was also used to explore the relationship between the brain connectivity of regions influenced by early exposure to pain stimuli and the NICI score. Fisher-transformed correlation coefficient values were considered as the metric of functional connectivity. All group analyses were controlled for mean frame wise displacement, gestational age at birth, gender and postmenstrual age at MRI. Results were thresholded at a voxel-wise *p* < 0.001 (FWE-corrected) two-sided level and cluster-level *p* < 0.05 (FWE-corrected) level.

### Brain Connectivity and Neurodevelopment

**FIGURE 1:**
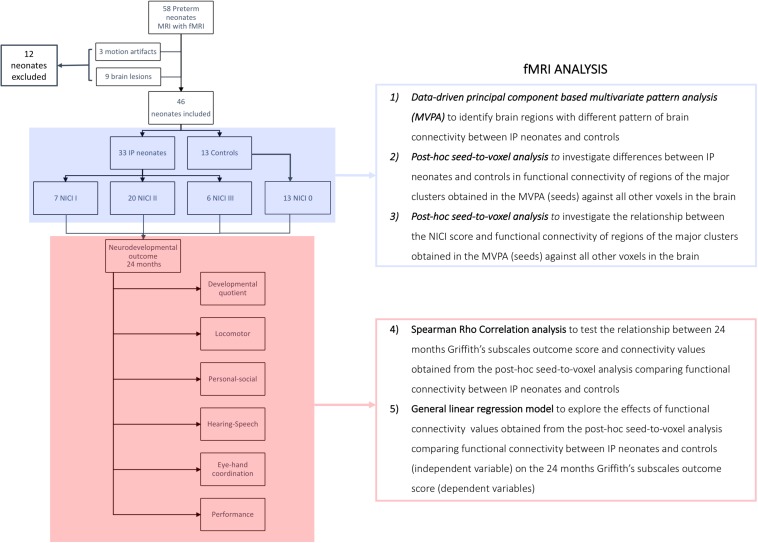
Flow-chart summarizing the fMRI analysis. Control group, neonates not requiring invasive procedures; IP group, neonates requiring invasive procedures; NICI score, neonatal intensive care invasiveness score; *0 category*, no painful events during the early post-natal period or <5 skin breaks (i.e., heel lances, central line insertion, and intramuscular injection); *I category*, >5 skin breaks or neonatal endotracheal intubation; *II category*, >5 skin breaks and neonatal endotracheal intubation; *III category*, surgical interventions (i.e., patent ductus arteriosus ligation and surgical necrotizing enterocolitis).

We evaluated the relationship between the brain connectivity of regions influenced by early pain exposure and the neurodevelopmental outcome at 24 months of corrected age, extracting connectivity values from the suprathreshold clusters obtained at the previous *post hoc* seed-to-voxel analysis. An exploratory analysis was conducted to test the relationship between these extracted functional connectivity values and outcome scores using a Spearman Rho correlation test. Subsequently, the effects of functional connectivity (independent variable) on the neurodevelopmental outcome scores at 24 months corrected-age (dependent variables) were examined using a general linear regression model. Variables known to influence brain development and clinical outcome were entered as covariates, including gestational age at birth and MRI (weeks), gender, total morphine dose, and days of intubation. Preliminary checks were conducted to ensure that there was no violation of the assumptions of normality, linearity, homogeneity of variances, homogeneity of regression slopes, and reliable measurement of the covariate (Figure 1).

Statistical analyses were performed using SPSS Statistics software, v21 (IBM, Armonk, NY, United States).

## Results

### Clinical Characteristics

Brain MRI studies of 112 consecutive preterm neonates were retrospectively evaluated. The BOLD fMRI sequence was performed in 58 neonates. The MRI studies of 12/58 patients (20.7%) were excluded: brain lesions were present in 9 cases (8 GMH-IVH, 2 arteriovenous malformations, and 2 brain malformations), and fMRI sequences were affected by motion artifacts in the remaining 3 neonates. Thus, the MRI studies of 46/58 (79.3%) preterm neonates were included in the study. Thirty-three (71.7%, 33/46) neonates were subjected to painful procedures during the neonatal intensive care (IP-group): 7/33 (21%) were assigned to the NICI I category, 20/33 (60%) to the NICI II category, and 6/33 (19%) to the NICI III category. Conversely, 13 preterm neonates (28.3%, 13/46) required less than 5 procedures during the perinatal period (control group) (Table 1).

**TABLE 1:** Demographic and clinical characteristics of neonates divided according to the NICI score categories.

	**Control-group**	**IP-group**			***P***
			
**NICI score**	**0**	**I**	**II**	**III**	
N° neonates	13	7	20	6	
Gestational age (week) *Median (range)*	31.2 (27.1–32.8)	28.2 (27.3–29.1)	27.1 (26.3–30.2)	27.9 (25.3–31.7)	0.085
Postmenstrual age at MRI (week) *Median (range)*	40.1 (39.1–41.2)	40.1 (39.3–40.3)	40.8 (38.9–41.3)	40.3 (39.4–41.9)	0.832
Birth weight (gr) *Median (range)*	1189.25 (863.81–1514.21)	1090.88 (844.32–1427.32)	1060.18 (713.41–1523.78)	1190.21 (756.54–1497.59)	0.743
Gender *N*°*neonates (%)*					0.833
Male	5 (38%)	4 (57%)	8 (40%)	3 (50%)	
Female	8 (62%)	3 (43%)	12 (60%)	3 (50%)	
APGAR 1’ *Median (range)*	8.81 (5–10)	6.97 (5–9)	6.43 (5–8)	6.58 (5–7)	0.011
APGAR 5’ *Median (range)*	9.15 (7–10)	8.91 (7–10)	8.21 (6–9)	7.22 (6–8)	0.003
Days of mechanical ventilation *Median (range)*	0 (0)	2 (0–4)	6 (2–15)	9 (3–21)	<0.001
Morphine dose^∗^ *Median (range); N*° *neonates*	0 (0); 0	0.15 (0.13–0.19); 4	1.8 (0.51–3.9); 16	2.93 (0.6–10.5); 6	<0.001
Midazolam dose^∗^ *Median (range); N*°*neonates*	0 (0); 0	1.2 (1–3.5); 4	2.3 (1.5–6.7); 7	6.5 (3.9–11.7); 5	<0.001
Dexamethasone^∗^ dose *Median (range); N*°*neonates*	0 (0); 0	0.94 (0.8–1.2); 3	0.88 (0.7–1.2); 7	1.2 (0.75–2.1); 4	<0.001
Total skin breaks *Median (range)*	1 (1–3)	9 (5–12)	13 (7–19)	28 (9–41)	<0.001
Endotracheal intubation *N*°*neonates (%)*	0 (0%)	0 (0%)	20 (100%)	6 (100%)	<0.001
Surgery *N*°*neonates (%)*	0 (0%)	0 (0%)	0 (0%)	6 (100%)	<0.001

*P indicates significance level of statistic comparisons. Continuous and categorical measures were compared using the Kruskall-Wallis and X^2^ tests, respectively. Control group, neonates not requiring invasive procedures; IP group, neonates requiring invasive procedures. NICI score, neonatal intensive care invasiveness score. ^∗^ Cumulative dose in milligrams adjusted for daily body weight (Kg).*

### Neurodevelopmental Outcome

Table 2 summarize results of neurodevelopmental outcome evaluation at 24 months. An abnormal neurodevelopmental outcome occurred more frequently in neonates who required significant invasive procedures in the neonatal period compared to the control group (*P* = 0.001). In particular, 20/46 (43.4%) ex-preterm children (all belonging to the IP group) obtained neurodevelopmental scores lower than 85 in at least one of the 6 developmental domains of the Griffiths scale: 3/7 (42.9%) of the NICI I category neonates, 12/20 (60%) of the NICI II category neonates, and 5/6 (83.3%) of the NICI III category neonates. Conversely, the performance of the remaining 26/46 (56.6%) children, including 13 IP neonates and all control children, was higher than 85 in all domains (Supplementary Figure S1).

**TABLE 2:** Clinical outcome at 24 months and NICI score categories.

**NICI score**		**Griffith’s developmental scale scores (mean ± std.dev)**					
		
		**Developmental quotient**	**Locomotor**	**Personal-social**	**Hearing-speech**	**Eye-hand coordination**	**Performance**
Control group	0 category *N* = 13	101.3 ± 3.1	101.5 ± 3.2	98.9 ± 4.2	101.9 ± 3.6	101.9 ± 3.7	99.9 ± 3.1
IP group	I category *N* = 7	98.4 ± 10.1	99.1 ± 5.7	90.1 ± 9.2	94.2 ± 9.6	91.3 ± 8.4	94.7 ± 7.2
	II category *N* = 20	90.2 ± 10.9	93.1 ± 11.2	87.6 ± 9.3	94.1 ± 7.4	90.1 ± 9.2	92.5 ± 8.5
	III category *N* = 6	87.8 ± 8.3^∗^	84.5 ± 5.3^∗^	83.5 ± 8.3^∗^	86.8 ± 4.6^∗^	86.7 ± 4.1^∗^	87.8 ± 3.1^∗^

*Significant lower values were observed for each subscale score in neonates of III Invasiveness score category when compared with controls. ^∗^ indicate *P* < 0.001 (Bonferroni corrected); differences of Griffith’s developmental subscale score mean values were testes among the NICI score categories using a general linear regression model, controlling for gestational age and gender. Control group, neonates not requiring invasive procedures; IP group, neonates requiring invasive procedures; NICI score, neonatal intensive care invasiveness score: *0 category*, no painful events during the early post-natal period or <5 skin breaks (i.e., heel lances, central line insertion, and intramuscular injection); *I category*, >5 skin breaks or neonatal endotracheal intubation; *II category*, >5 skin breaks and neonatal endotracheal intubation; *III category*, surgical interventions (i.e., patent ductus arteriosus ligation and surgical necrotizing enterocolitis).*

### Functional Brain Connectivity

**FIGURE 2:**
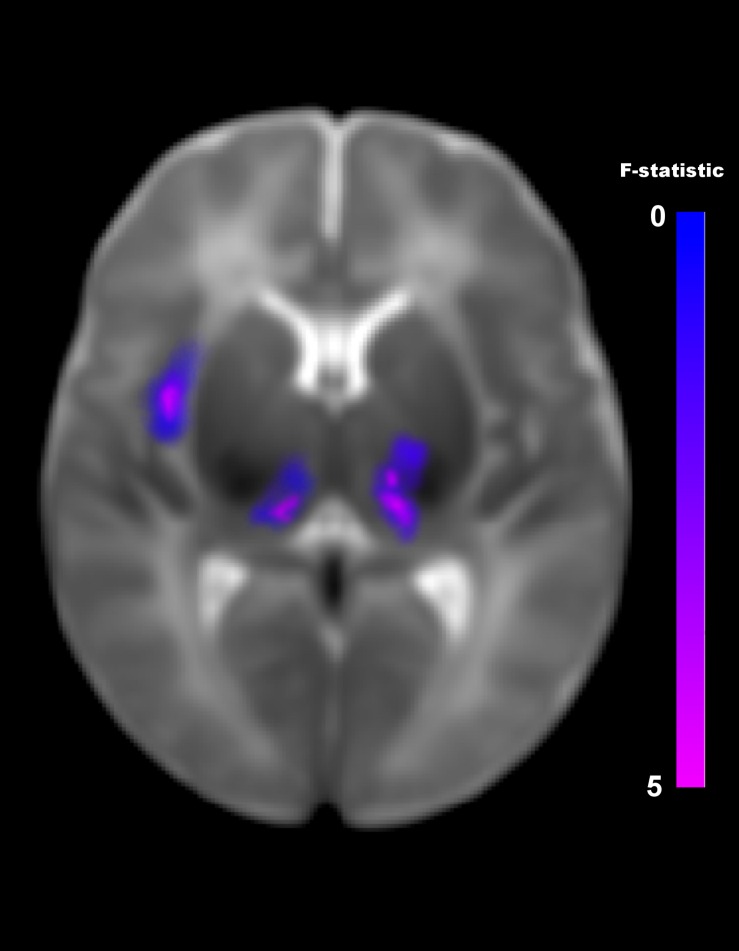
Differences in brain connectivity profile indexed at a voxel level using multivariate pattern analysis (MVPA). MVPA results are overlaid on a representative neonatal axial T2-weighted image. Blue-violet clusters indicates brain regions (thalami and right insular cortex) that show different pattern of functional connectivity between IP neonates and controls. Colorbar indicates F-statistic of between group differences with regard to the spatial maps of the four principal components. Three clusters were identified: in both thalami and in the right insula. The F-maps are threshold voxelwise at a *p* < 0.001 FWE-corrected and at *p* < 0.05 FWE-corrected at cluster-level.

**FIGURE 3:**
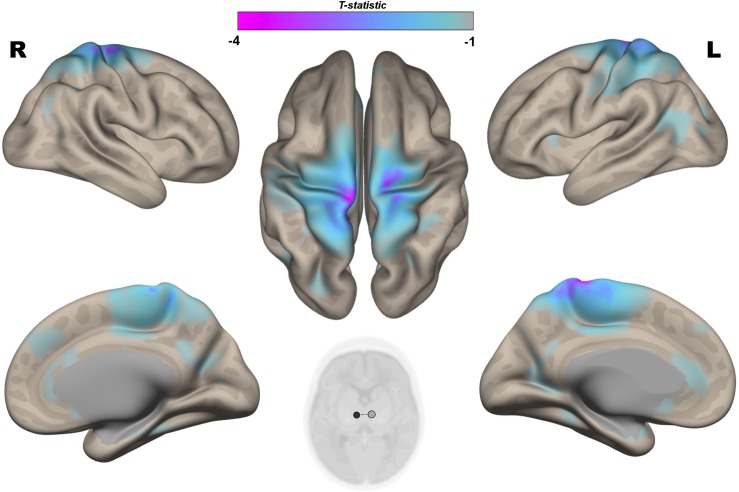
*Post hoc* Seed-to-Voxel analysis using seed regions at level of thalami at the peak coordinates from MVPA. This analysis identified decreased functional connectivity between thalami and somatosensory areas (blue-violet clusters) in the IP neonates when compared with controls. Colorbar indicates T-statistic of between groups differences (IP neonates < controls), the T-map is thresholded at voxelwise *p* < 0.001 FWE-corrected, and *p* < 0.05 FWE-corrected clusters level and at voxel level.

**FIGURE 4:**
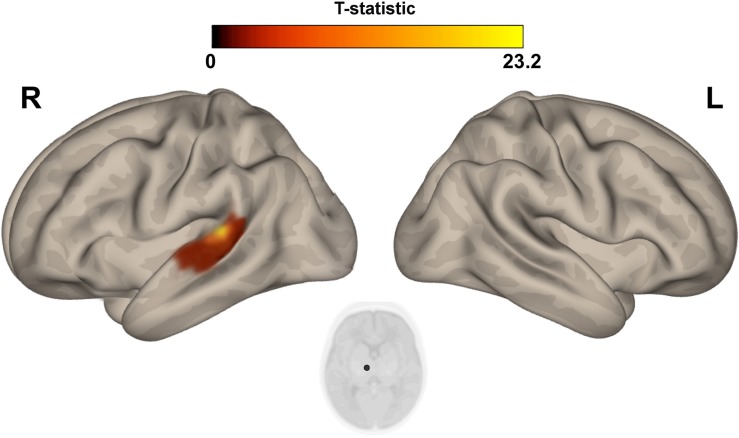
*Post hoc* Seed-to-Voxel analysis using seed regions at level of thalami at the peak coordinates from MVPA. This analysis identified increased functional connectivity between right thalamus and ipsylateral superior temporal gyrus (red-yellow clusters) in the IP neonates when compared with controls. Colorbar indicates T-statistic of between groups differences (IP neonates > controls), the T-map is thresholded at voxelwise *p* < 0.001 FWE-corrected and *p* < 0.05 FWE-corrected clusters level and at voxel level.

The multivariate pattern analysis analysis showed group differences with regard to whole brain functional connectivity patterns in three regions: the right insular cortex (rIC) and right and left thalami (Figure 2). In the *post hoc* seed correlation analyses, performed using seed regions based on the MVPA results, we found decreased connectivity in the IP group compared to control neonates between both thalami and bilateral somatosensory regions (pre-central and post-central gyri) (Figure 3), while we found stronger connectivity between right thalamus and the right superior temporal gyrus (Figure 4).

**FIGURE 5:**
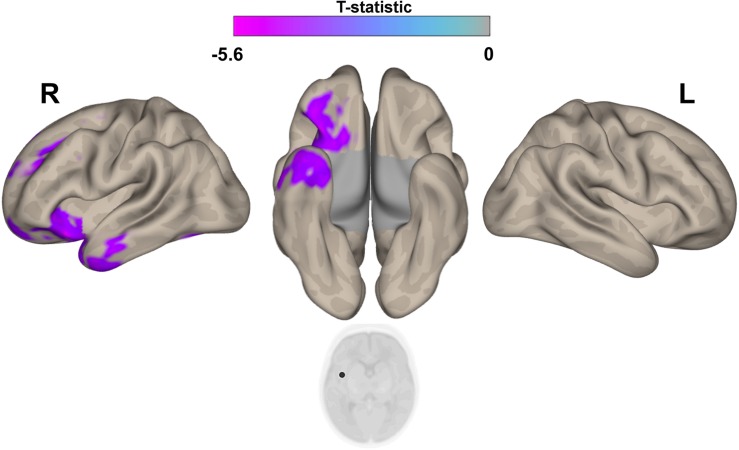
*Post hoc* Seed-to-Voxel analysis using seed regions at level of right insular cortex at the peak coordinates from MVPA. This analysis identified decreased functional connectivity between right insular cortex and ipsilateral amygdala/hyppocampus (blue-violet clusters) in the IP neonates when compared with controls. Colorbar indicates T-statistic of between groups differences (IP neonates < controls), the T-map is thresholded at voxelwise *p* < 0.001 FWE-corrected and *p* < 0.05 FWE-corrected clusters level and at voxel level.

The *post hoc* seed-to-voxel analysis performed from the seed region of right insular cortex revealed a weaker connectivity with the right amygdala and the right hippocampal and parahippocampal regions (Figure 5 and Table 3).

**TABLE 3:** Group differences in functional connectivity.

**Contrast**	**Seed (center of sphere)**	**Target (peak coordinates)**	**Cluster size (# voxels)**	**Cluster p-FWE**
Controls > IP	Left thalamus (−7, −15,2)	Left Somatosensory (−4, −27, 34)	1873	<0.000001
		Right Somatosensory (6, −25, 42)	973	0.00006
	Right thalamus (8, −18, 5)	Right Somatosensory (5, −26, 37)	2373	<0.000001
		Left Somatosensory (−11, −26, 42)	665	0.00008
	Right insula (26, −2, 4)	Right amygdala/hippocampus (14, −10, 15)	871	0.00007
IP > Controls	Right thalamus (8, −18, 5)	Right superior temporal gyrus (40, −19, 3)	672	0.00027

*Seed regions were defined *post hoc* based on results from the MVPA analysis. Target regions are labeled based on the locations of the largest number of voxels within significant cluster. All results are significant on a corrected cluster level (*p* < 0.0005 FDR). Controls, non-invasive procedure group; IP, invasive procedure group.*

The correlation analysis between the NICI score and the functional connectivity revealed a significant negative correlation between the NICI score and the connectivity strength in the right thalamus and ipsilateral somatosensory cortex, indicating weaker functional connectivity in thalamocortical circuits in neonates who experienced a greater number of invasive procedures (*r* = −0.651; *P* = 0.0003).

### Functional Brain Connectivity and Neurodevelopmental Outcome

**FIGURE 6:**
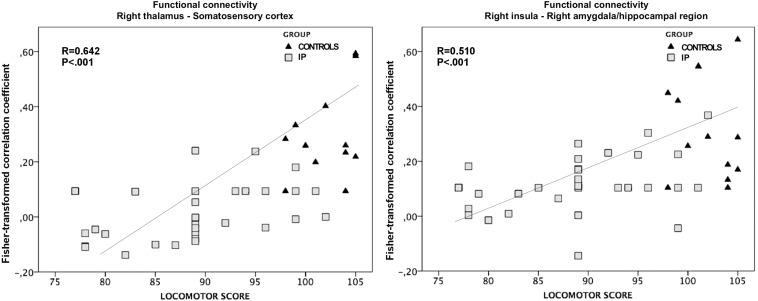
The association between altered functional connectivity and locomotor subscale score. The strength of functional connectivity between the right thalamus and somatosensory cortex and between the right insular cortex and the ipsilateral amygdala and hippocampus correlates positively with neonates’ performance on the locomotor task (*r* = 0.642; *P* < 0.001 and *r* = 0.510; *P* < 0.001, respectively).

The exploratory correlation analysis revealed a significant positive correlation between the locomotor subscale scores and the functional connectivity between the right thalamus and ipsilateral somatosensory cortices (*r* = 0.642; *P* < 0.001). We also observed a positive correlation between the locomotor score and the functional connectivity between the right insula and the ipsilateral amygdala and hippocampal/parahippocampal areas (*r* = 0.510; *P* < 0.001) (Figure 6). No significant correlations were observed between the functional connectivity of these regions and other neurodevelopmental subscale scores, nor between the functional connectivity of the left thalamus and all neurodevelopmental scores.

The general linear regression analyses exploring the effects of functional connectivity of the right thalamus and right insula at term equivalent age on the locomotor outcome at 24 months of corrected age yielded the following results: the locomotor score was predicted by the functional connectivity of right thalamus and perirolandic cortex (ß = 23.506; *P* = 0.003), while the functional connectivity of the right insula was not a significant predictor of the locomotor outcome (ß = 15.785; *P* = 0.085).

## Discussion

This fMRI study unravels altered brain functional connectivity at term equivalent age in preterm neonates who experienced significant painful events during neonatal intensive care, and demonstrates a relationship of these fMRI abnormalities with the motor outcome at 24 months of corrected age. In particular, in these neonates we demonstrated weaker functional connectivity in the thalamocortical pathways and between the right insula and limbic system. Of note, the thalamic nuclei, thalamocortical pathways and limbic structures play a crucial role in encoding noxious stimuli (Caulo et al., 2019). More in detail, the thalamus receives nociceptive signals via two major ascending pathways: the spinothalamic and spino-reticulo-thalamic tracts. The spinothalamic tract conveys information about noxious stimuli from the dorsal horn and spinal trigeminal nucleus to both the lateral and medial thalamus. In contrast, the spino-reticulo-thalamic tract is thought to carry nociceptive information specifically to the medial thalamus via an additional synaptic relay within the medullary reticular formation of the brainstem (Gauriau and Bernard, 2002). These thalamic regions innervate specific sets of cortical target areas, including the somatosensory and limbic cortices (Groh et al., 2018), with different functions of thalamocortical pathways in the regulation of both sensory-discriminative and emotional aspects of pain stimuli. The transmission of pain impulses to cerebral cortex and subcortical gray matters represents an important requirement for the descending modulation of the spinal response to painful events (Groh et al., 2018). Of note, this nociceptive circuitry is functionally immature in neonates, especially during the early post-natal period (Fitzgerald, 2005).

Due to the immaturity of the descending modulation of nociceptive activity (Hatfield, 2014), preterm neonates may demonstrate central sensitization to repeated painful stimuli, which may in turn alter the brain microstructure and production of stress hormones, with poorer cognitive, motor, and behavioral neurodevelopment (Fitzgerald et al., 1989; Walker et al., 2009b).

Interestingly, previous studies examining the effects of early exposition to painful events on brain development in preterm neonates showed that neonatal pain was associated with decreased brain volume particularly impacting frontal and parietal lobes at term equivalent age (Smith et al., 2011; Brummelte et al., 2012). More recently, Duerden et al. demonstrated that also subcortical structures may be vulnerable to procedural pain corroborating the original finding of impaired white matter and subcortical gray matter with early pain exposure of premature babies. In particular, they found that preterm neonates requiring invasive procedures during the early postnatal period showed decreased thalamic NAA/Cho and microstructural alterations in thalamocortical pathways. Remarkably, these structural and metabolic thalamic abnormalities correlated with poorer cognitive and motor outcomes (Duerden et al., 2018).

Our findings confirm the association between early invasive procedures and altered development of the thalamus and thalamocortical pathways in preterm neonates. Indeed, we speculate that weaker functional connectivity observed in IP neonates may derive from the impaired development of thalami and thalamocortical connections during the early postnatal period of very premature babies. Of note, during brain development, maturation and topographic organization of afferent thalamocortical projections occur through an activity-dependent manner (Shatz, 1990; Goodman and Shatz, 1993), with axons from the thalamus contending for representation in the cortex in response to both external stimuli and endogenous NMDA-dependent mechanisms (Crair and Malenka, 1995). Abnormal amplified external stimuli related to pain may disrupt the activity of this pathway during early brain development, and may determine abnormal distribution of thalamocortical projections and atypical somatosensory cortex development.

Recently, it has been demonstrated that early procedural pain induces a greater release of glutamate, since NMDA receptors involved in the transmission of pain signals are more active during early life (Vinall and Grunau, 2014). Excessive release of glutamate may thus determine further trigger to both oxidative stress and inflammatory reactions in the premature neonatal brain (Panfoli et al., 2018), that may arrest the development of subplate neurons and preoligodendrocytes, which are particularly vulnerable to reactive oxygen, nitrogen species, and cytokines secreted by microglia (Back et al., 1998; Haynes et al., 2003; Slater et al., 2012; Panfoli et al., 2018; Tortora et al., 2018a, b). Taken together, these data suggest that repeated exposure to neonatal procedural pain is associated with alterations of both neuronal structure and function with potential relevant consequences onto neurodevelopmental outcome (Boardman et al., 2010; Chau et al., 2013; Duerden et al., 2018).

Another important result of our study was the weaker functional connectivity between the insular cortex and the limbic structures (i.e., amygdala and hyppocampus) in IP neonates. Interestingly, the limbic system and hypothalamus, together with several other cortical and subcortical brain regions, are fundamental in creating a descending feedback loop that modulates spinal dorsal horn activity in response to external stimuli (Beggs, 2015). In particular, the segregation of afferent inputs to the spinal cord from the peripheral central nervous system allows to distinguish sensory information as tactile, thermal, or nociceptive. This process occurs postnatally and requires several modulation processes at different levels of the central nervous system. The dorsal horn of the spinal cord is the first point of modulation of sensory information from the periphery, acting as a site of complex integration of sensorial information. In addition to this local circuitry, brain modulatory networks including the limbic system and hypothalamus regulate the spinal activity through both facilitatory and inhibitory inputs (Beggs, 2015). In early postnatal life, these descending pathways are almost exclusively facilitatory and targeted to low-threshold (tactile) input, promoting an ongoing activity-dependent maturation of dorsal horn nociceptive circuitry. On the other hand, the descending inhibitory control, which is pivotal for modulating pain perception being selectively targeted to high-threshold (painful) stimuli, develops later (Koch and Fitzgerald, 2014). Here, we postulate that the lower functional connectivity observed between the insular cortex and limbic system in IP neonates may reflect abnormal maturation of these cortical networks due to the exposure to repeated painful stimuli during the early postnatal period, particularly in the premature brains.

Finally, fMRI analysis demonstrated a relationship between connectivity alterations of the thalamus and insular cortex and specific neurodevelopmental impairments at 24 months of age. In particular, weaker intrinsic functional connectivity of the thalamocortical pathways, and the insular cortex-limbic connections was associated with poorer locomotor outcome. This finding is corroborated by prior reports demonstrating that preterm neonates exposed to early postnatal painful events showed macro- and microstructural alterations in thalamocortical projections and other cortical regions (i.e., frontal and parietal cortex) that were associated with adverse motor and cognitive neurodevelopmental outcome in the first years of life, involving also the mechanisms of anxiety and emotion regulation (Duerden et al., 2015; Tortora et al., 2015; Navarra et al., 2016; Malavolti et al., 2017; Filippa et al., 2019). Moreover, Duerden et al. (2018) demonstrated that early pain exposure can influence thalamocortical pathways maturation, especially in extremely preterm born neonates, and that the thalamic volume growth during the first weeks of life correlated with cognitive and motor scores at 36 months of age. Intriguingly, we found that functional connectivity of thalamocortical pathways remains the single factor able to predict the locomotor outcome at 24 months of age after controlling for the confounding effects of the clinical factors, thus supporting the important role of thalami in the development of motor functions in preterm neonates (Lao et al., 2016). Further studies on cognitive functions and emotional processing of preterm neonates studied at school age are necessary to elucidate whether these functional connectivity changes may impact also on academic or social/behavioral performances.

## Limitations

Limitations of this study include the relatively small sample size, albeit justified by the small incidence of normal MRI examination in very early preterm neonates. Moreover the short neurodevelopmental follow-up performed at 24 months of corrected age, the lack of information about the socio-economic status and educational level of parents, and the use of a limited numbers of clinical confounders in the fMRI analysis could represent a source of bias. In addition, the difficulty of distinguishing between direct effects of exposure to painful stimuli and the confounding clinical and demographic variables may play a role. In this study, we considered the confounding effects of gestational age, gender, age at MRI scan and the length of stay in the neonatal intensive care unit. Moreover, we included in the analysis several clinical factors, such as total morphine and days of intubation, which may have influenced functional brain development, the degree of neuroinflammation, and clinical outcome. Finally, we selected only patients with normal brain MRI to avoid the confounding effect of brain lesions on functional connectivity.

## Conclusion

In summary, our findings suggest that early exposure to pain is associated with abnormal functional connectivity of developing networks involved in the modulation of noxious stimuli in preterm neonates, contributing to the neurodevelopmental consequence of preterm birth. Future follow-up studies are needed to determine how the consequences of early painful events may affect the late developmental outcome within groups of babies of the same gestational age and how it can be mitigated by appropriate pharmacological analgesia or by other positive systematic sensorial stimulation known to reduce nociceptive reaction of preterm neonates (Ramenghi et al., 1999).

## Data Availability

The raw data supporting the conclusions of this manuscript will be made available by the authors, without undue reservation, to any qualified researcher.

## Ethics Statement

The local ethics committee (Comitato Etico Regione Liguria, Genoa, Italy) approved this study and parents provided written informed consent in accordance with the Declaration of Helsinki.

## Author Contributions

DT analyzed and interpreted the data, and drafted the manuscript. MS, MM, CD, AP, DM, CT, SU, LB, and GM collected the data and revised the manuscript. AR and LR designed the study, interpreted the data, and revised the manuscript. All authors discussed the results and commented on the manuscript.

## Conflict of Interest Statement

The authors declare that the research was conducted in the absence of any commercial or financial relationships that could be construed as a potential conflict of interest.

## Footnotes

1 http://brainsuite.org

2 http://www.nitrc.org/projects/conn
