# Supplementary material for: Early Pain Exposure Influences Functional Brain Connectivity in Very Preterm Neonates
Source: Front Neurosci. 2019 Aug 23;13:899. doi: 10.3389/fnins.2019.00899 (PMC6716476; doi:10.3389/fnins.2019.00899)
Supplement: Supplementary file 1 [file Data_Sheet_1.PDF]

Online-figure1

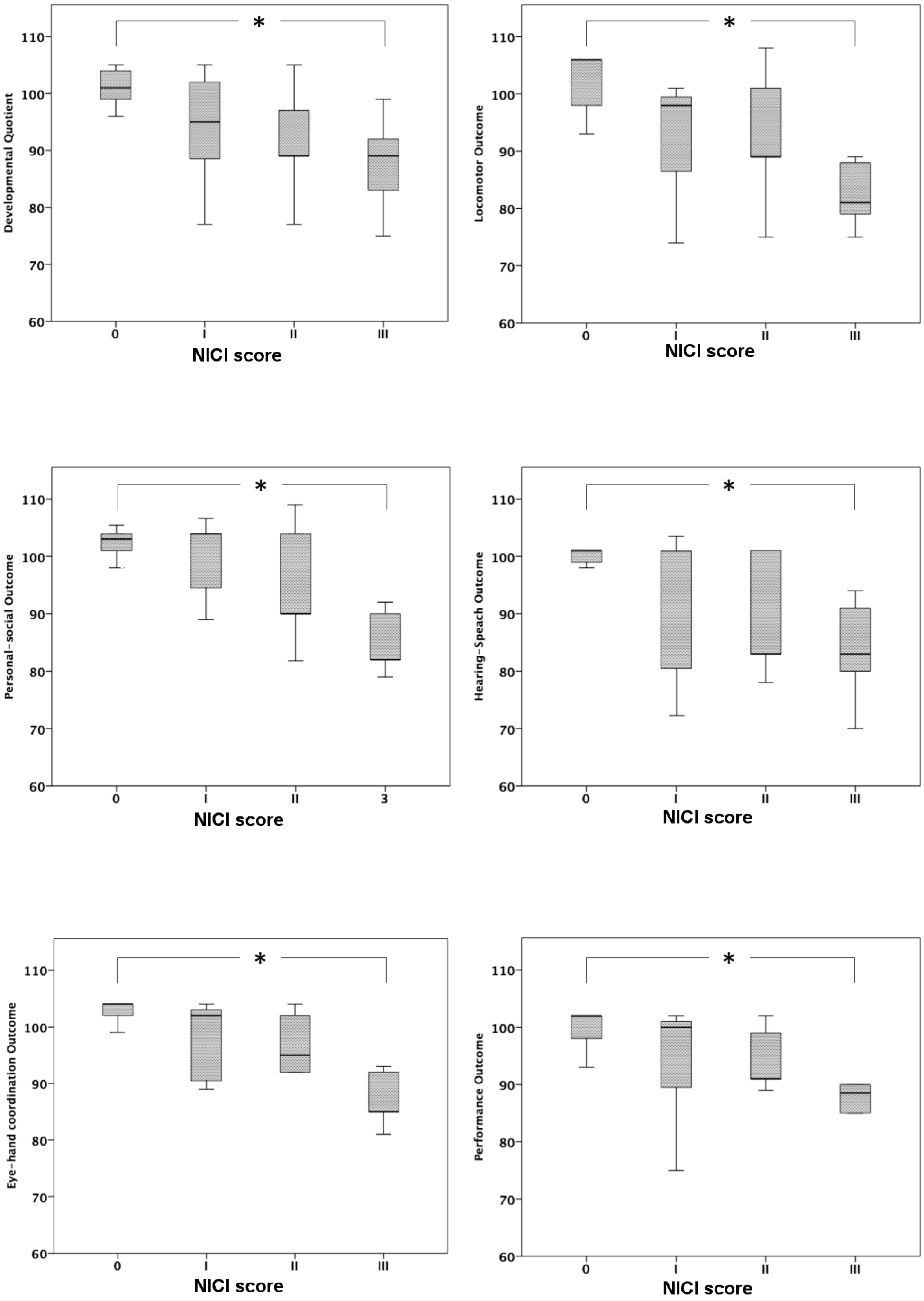

Online-Figure 1. Relationship between the Neurodevelopmental Griffith's subscale scores and the Neonatal Intensive Care Invasiveness (NICI) score categories. Note that Neurodevelopmental scores are significantly different in the four NICI categories, especially when comparing Controls (0 category) and neonates that experienced the highest number of invasive

procedures (III category). Note that **0** indicates no painful events during the early post-natal period or <5 skin breaks (i.e. heel lances, central line insertion, intramuscular injection); **I**, >5 skin breaks or neonatal endotracheal intubation; **II**, >5 skin breaks and neonatal endotracheal intubation; **III**, surgical interventions (i.e. patent ductus arteriosus ligation, surgical necrotizing enterocolitis).

* indicates P=.01.
